# Parents’ and guardians’ views on the acceptability of a future COVID-19 vaccine: A multi-methods study in England

**DOI:** 10.1016/j.vaccine.2020.10.027

**Published:** 2020-11-17

**Authors:** Sadie Bell, Richard Clarke, Sandra Mounier-Jack, Jemma L. Walker, Pauline Paterson

**Affiliations:** a Department of Global Health and Development, Faculty of Public Health and Policy, London School of Hygiene & Tropical Medicine, 15-17 Tavistock Place, London WC1H 9SH, UK; b Newcastle University Business School, Newcastle University London, 102 Middlesex Street, London E1 7EZ, UK; c Department of Infectious Disease Epidemiology, Faculty of Epidemiology and Population Health, London School of Hygiene & Tropical Medicine, Keppel Street, London WC1E 7HT, UK; d Department of Statistics, Modelling and Economics, Public Health England, National Infection Service, 61 Colindale Ave, London NW9 5EQ, UK

**Keywords:** COVID-19, Coronavirus, Vaccine, Acceptance, Child

## Abstract

**Background:**

The availability of a COVID-19 vaccine has been heralded as key to controlling the COVID-19 pandemic. COVID-19 vaccination programme success will rely on public willingness to be vaccinated.

**Methods:**

We used a multi-methods approach - involving an online cross-sectional survey and semi-structured interviews - to investigate parents’ and guardians’ views on the acceptability of a future COVID-19 vaccine. 1252 parents and guardians (aged 16 + years) who reported living in England with a child aged 18 months or under completed the survey. Nineteen survey participants were interviewed.

**Findings:**

Most survey participants reported they would likely accept a COVID-19 vaccine for themselves (Definitely 55.8%; Unsure but leaning towards yes 34.3%) and their child/children (Definitely 48.2%; Unsure but leaning towards yes 40.9%). Less than 4% of survey participants reported that they would definitely not accept a COVID-19 vaccine. Survey participants were more likely to accept a COVID-19 vaccine for themselves than their child/children. Participants that self-reported as Black, Asian, Chinese, Mixed or Other ethnicity were almost 3 times more likely to reject a COVID-19 vaccine for themselves and their children than White British, White Irish and White Other participants. Survey participants from lower-income households were also more likely to reject a COVID-19 vaccine. In open-text survey responses and interviews, self-protection from COVID-19 was reported as the main reason for vaccine acceptance. Common concerns identified in open-text responses and interviews were around COVID-19 vaccine safety and effectiveness, mostly prompted by the newness and rapid development of the vaccine.

**Conclusion:**

Information on how COVID-19 vaccines are developed and tested, including their safety and efficacy, must be communicated clearly to the public. To prevent inequalities in uptake, it is crucial to understand and address factors that may affect COVID-19 vaccine acceptability in ethnic minority and lower-income groups who are disproportionately affected by COVID-19.

## Highlights

• Most parents stated they would likely accept a COVID-19 vaccine for themselves and their children.
• Ethnicity and household income were predictors of COVID-19 vaccine refusal.
• The main motivation for vaccine acceptance was for self-protection against COVID-19.
• Foremost concerns were around the safety and efficacy of a ‘rushed’ new vaccine.

## 1 Introduction

Over 33 million confirmed cases of coronavirus (COVID-19), including more than 1 million deaths, have been reported globally since the start of the COVID-19 pandemic to 29th September 2020 [1]. COVID-19 has spread to almost every country in the world, resulting in excess mortality and serious social and economic disruptions.

According to COVID-19 mortality figures, the UK is one of the worst affected countries in the world, with over 440,000 cases and 42,000 deaths reported as of 29th September 2020 [1], [2]. In efforts to slow the spread of COVID-19 in the UK, stringent restrictions on movement were enforced on 23rd March 2020, with the general public directed to only leave their homes to: shop for basic essentials, take one form of exercise per day, access medical care or help a vulnerable person, and to travel to and from work if absolutely necessary (where unable to work from home) [3]. People most at-risk from COVID-19 were asked to protect themselves by shielding from 23rd March 2020: staying at home at all times, for at least 12 weeks. In addition, schools were closed to all but vulnerable children and children of key workers [3].

Although restrictions to slow COVID-19 spread have eased in many countries, including the UK, day-to-day life globally remains distinctly marked by the COVID-19 pandemic, and measures to prevent COVID-19 spread [4]. A COVID-19 vaccine has been heralded as key to ending the pandemic and clinical trials to develop a COVID-19 vaccine are currently being conducted at an accelerated rate [5], [6]. To date, over 160 COVID-19 vaccine candidates are under development, with 26 in human trials [6]. In the UK, a Vaccine taskforce has been created to ‘drive forward, expedite and co-ordinate efforts to research and then produce a coronavirus vaccine and make sure one is made available to the public as quickly as possible’ [5]. On 20th July 2020, the UK government secured early access to 90 million doses of promising COVID-19 vaccines, ‘giving the UK the most likely chance of getting access to a safe and effective vaccine at the quickest speed’ [7].

In planning for the near-future availability of a COVID-19 vaccine, as well as focusing on how to deliver COVID-19 vaccine programmes and ensure equitable vaccine allocation globally [8], it is crucial to explore the acceptability of a COVID-19 vaccine to the public. The success of any COVID-19 vaccination programme will depend on public willingness to receive the vaccination.

In the UK, the Joint Committee on Vaccination and Immunisation (JCVI), have provisionally advised that if and when a COVID-19 vaccine becomes available the priority should be to vaccinate those at highest risk of COVID-19 exposure, transmission and severe COVID-19 disease [9]. The JCVI has communicated a preliminary list of priority groups to receive the vaccine that targets those most at risk of disease although it recognises that depending on future vaccine characteristics, it may be that vaccinating young and healthy members of the population will help to prevent COVID-19 transmission to high-risk groups and also reduce societal and economic disruption.

In recognition that a COVID-19 vaccine could be made eligible to the majority of the population, we conducted a multi-methods study to investigate the views of parents and guardians in England on the acceptability of a future COVID-19 vaccine, for themselves and their children.

## 2 Methods

We used a multi-methods approach – combining qualitative and quantitative methods – with the aim of gaining a more complete insight into the acceptability of a future COVID-19 vaccine. The study utilised a cross-sectional questionnaire survey and semi-structured interviews, to quantify the prevalence of different views on COVID-19 vaccine acceptability, and to explore reasons behind these views. The questions asked regarding COVID-19 vaccine acceptance were part of a larger study exploring parents’ and guardians’ views and perceptions towards childhood vaccinations and use of NHS general practice services for childhood vaccination during the COVID-19 pandemic

## 3 Cross-sectional survey

### 3.1 Recruitment

Survey recruitment took place between 19th April and 11th May 2020. Eligible participants were required to be (1) a parent or guardian of a child (or children) aged 18 months or under, (2) a resident of England, and (3) aged 16 + years. Participants were provided with full study details before starting to complete the survey (see supplemental file 1), explaining all aspects of participation including their right to withdraw from the research and issues surrounding confidentiality and data protection. Before completing the survey, participants were required to indicate their consent through the use of a forced response question. It was made clear that only the recorded data from participants that completed the full survey would be used in later analysis.

We utilised an online social media strategy to recruit survey participants. This strategy involved the creation of a Facebook page dedicated to the study with a single post advertising the study, outlining the eligibility criteria for participation, and including the hyperlink to the survey. An email was then sent to organisers of 284 baby and toddler play groups in England. This contact email included a study summary and a request to share the advertising post through the groups various means of communication (e.g. Facebook, WhatsApp, Twitter or an email list). In addition to this, Facebook’s paid promotion feature was used to target the survey at eligible potential participants.

### 3.2 Survey design and pre-testing

The survey was developed by the study researchers in consultation with immunisation representatives from Public Health England. In the refinement of the survey questions and layout, we gained feedback from parents with young children on the comprehensibility, usability and time taken to complete the survey from the perspective of the target audience.

### 3.3 Measures

The survey included demographic questions concerning age, gender, household income, employment, marital status of participants, and number and age of children. Two questions were designed to capture acceptance of a new COVID-19 vaccination. The text of the questions read “*If a new coronavirus (COVID-19) vaccine became available would you accept the vaccine for yourself?*” and “*If a new coronavirus (COVID-19) vaccine became available would you accept the vaccine for your child/children?*”. A 4-point Likert scale was used to encourage participants to take a stance on the vaccine (rather than selecting a ‘do not know’ answer). The Likert scale options were *“Yes, definitely”*, *“Unsure but leaning towards yes”*, *“Unsure but leaning towards no”* and *“No, definitely not”*. Open-text boxes were included for participants to explain their responses to the two questions on vaccine acceptability for themselves and for their children. Participants could give more than one reason in the open-text boxes.

### 3.4 Analysis

A paired samples *t*-test was used to compare acceptance of a COVID-19 vaccine for self and for the participant’s child. Two subsequent logistic regressions were then conducted to determine the demographic factors associated with rejection of the COVID-19 vaccine for both self-vaccination and that of the participant’s child. Alpha was set to 0.05 for all tests. Age, household income, ethnicity, location, and employment were included as predictive variables in the logistic regression models. In the logistic regression model for child vaccination, number of children was also included as a predictive variable.

To perform the logistic regressions ethnicity was dichotomised into *‘White’* (i.e. White British, White Irish and White Other participants) and *‘Black, Asian or minority ethnic (BAME)’* (i.e. Black, Asian, Chinese, Mixed or Other ethnicity). Household income brackets were reduced to a *“low income”* (<£35,000), *“medium income”* (£35,000 - £84,999) and *“high income”* (>£85,000), and the vaccine acceptance variables were dichotomised into *“likely to accept”* (those that answered *Yes, definitely* or *Unsure but leaning towards yes*) and *“likely to reject”* (those that answered *No, definitely no*t or *Unsure but leaning towards no*).

Open text responses were analysed thematically in Microsoft Excel by PP. Coding schemes were produced based on the content of the open-text comments and discussed by PP and SB.

### 3.5 Semi-structured interviews

On completing the survey, participants were asked to provide their contact details if interested in taking part in a follow-up semi-structured interview. Participants that had expressed interest were purposively selected based on a range of characteristics, including ethnicity, household income, and geographical location. We purposefully aimed to interview survey participants who did not complete open text responses and presented characteristics of interest such as underrepresented populations in the survey (e.g. participants from ethnic minority groups or reporting a lower household income) and/or indicated they would likely refuse a COVID-19 vaccine, for their child or themselves. We did not solely interview participants who were likely to refuse a COVID-19 vaccine as we were keen to explore the nuances of reasons participants had for accepting or refusing a COVID-19 vaccine.

Participants were emailed an information sheet, fully detailing the study objectives and explaining all aspects of participation, including the right to withdraw from the research. Written informed consent was obtained from each participant. Interviews lasted approximately 30 min and were conducted over the phone. Topic guides, shaped around the content of the questionnaire, were used to assist the interviews. The interviews included two questions related to a COVID-19 vaccination, the first focused on parents’ and guardians’ views on receiving a new COVID-19 vaccine for themselves, should one become publicly available, and the second on their views on their child of accepting a new COVID-19 vaccine for their child. Interview participants received a £10 gift voucher as a thank you for their time and contribution. The interviews took place between 27th April and 27th May 2020.

Interviews were transcribed verbatim and analysed thematically in NVivo12 by using the stages outlined by Braun and Clarke [10]: data familiarisation, coding and theme identification and refinement. The transcribed interviews were read and coded by SB. To enhance the rigour of the analysis, coding approaches and subsequent theme generation and refinement was discussed between SB and the other researchers.

## 4 Findings

1252 parents and guardians completed the survey (see participant characteristics in Table 1). Most survey participants were female (95.0%; n = 1190), raising a child/children with a partner (97.0%; n = 1214), and identified as being White British, White Irish or White Other (94.1%; n = 1178). The age range of participants was 18–48 years (Mean = 32.95, SD = 4.565). Median household income was reported as £55,000-£64,999. Of the survey participants, 1049 (83.8%) left open-text responses to the open text survey questions.

**Table 1:** Characteristics of survey participants.

**Characteristic**		**Frequency (%)**
**Location**		
	South East	286 (22.8)
	Greater London	164 (13.1)
	North West	90 (7.2)
	East of England	231 (18.5)
	West Midlands	98 (7.8)
	South West	139 (11.1)
	Yorkshire and the Humber	116 (9.3)
	East Midlands	70 (5.6)
	North East	53 (4.2)
	Prefer not to answer	5 (0.4)

**Ethnicity**		
	White:- British	1082 (86.4)
	White:- Irish	20 (1.6)
	White:- Other white background	76 (6.1)
	Black or Black British:- African	3 (0.2)
	Black or Black British:- Caribbean	1 (0.1)
	Mixed:- White and Black Caribbean	7 (0.6)
	Mixed:- White and Black African	1 (0.1)
	Mixed:- White and Asian	9 (0.7)
	Mixed:- Other mixed background	7 (0.6)
	Asian or Asian British:- Indian	15 (1.2)
	Asian or Asian British:- Pakistani	10 (0.8)
	Asian or Asian British:- Bangladeshi	3 (0.2)
	Asian or Asian British:- Other Asian background	3 (0.2)
	Chinese	2 (0.2)
	Other ethnic group not represented by these options	7 (0.6)
	Do not wish to say	6 (0.5)

**Employment status**		
	Working full-time (over 30 h per week) or on parental leave from full time employment	679 (54.2)
	Working part-time (less than 30 h per week) or on parental leave from part time employment	404 (32.3)
	Homemaker	114 (9.1)
	Student	12 (0.9)
	Unemployed	13 (1.0)
	Other	23 (1.8)
	Prefer not to answer	7 (0.6)

**Household income (GBP)**		
	Under £34,999	255 (20.4)
	£35,000–£84,999	638 (51.0)
	£85,000 and over	267 (21.3)
	Prefer not to answer	92 (7.3)

**Number of children**		
	1	558 (44.6)
	2	504 (40.3)
	3	148 (11.8)
	4 or more	42 (3.4)

**Age of youngest child**		
	< 2 months	223 (18.0)
	3–5 months	330 (26.6)
	6–8 months	179 (14.4)
	9–11 months	154 (12.3)
	12–14 months	218 (17.6)
	>15 months	138 (11.1)

43.3% of survey participants (n = 530) provided their details to be contacted for a follow-on interview. In total, 61 parents were contacted to participate. Of these 19 took part in interviews (18 women and one man), 39 did not respond to recruitment emails, two responded initially but did not follow through with an interview. The characteristics of interviewees are outlined in Table 2.

**Table 2:** Characteristics of interview participants.

**No.**	**Age**	**Ethnicity**	**Region**	**Household Income**	**No. of children**	**Age of youngest child at time of interview**	**Response to: If a new coronavirus (COVID-19) vaccine became available would you accept the vaccine for your child/children?**	**Response to: If a new coronavirus (COVID-19) vaccine became available would you accept the vaccine for yourself?**
#1	25	Mixed:- White and Asian	South East	Under £34,999	1	13 wks	Unsure but leaning towards yes	Unsure but leaning towards yes
#2	33	White: - Other white background	South West	Prefer not to answer	4	~ 13 mos	Yes definitely	Yes definitely
#3	28	White British	East Midlands	£35,000–£84,999	2	~17 wks	Unsure but leaning towards yes	Yes definitely
#4	30	White British	South West	£85,000 and over	1	8.5 mos	Yes definitely	Yes definitely
#5	31	White British	Yorkshire and Humber	£35,000–£84,999	2	14 wks	Unsure but leaning towards yes	Unsure but leaning towards yes
#6	39	White British	South West	£35,000–£84,999	3	12 wks	Unsure but leaning towards yes	Unsure but leaning towards yes
#7	36	White British	Greater London	£85,000 and over	2	3 mos	Unsure but leaning towards yes	Unsure but leaning towards yes
#8	36	White: - Other white background	Greater London	£85,000 and over	2	15 wks	Unsure but leaning towards no	Unsure but leaning towards yes
#9	33	White British	South East	Under £34,999	2	14 mos	Yes definitely	Yes definitely
#10	34	Mixed:- White and Black Caribbean	West Midlands	£85,000 and over	1	9 wks	Unsure but leaning towards yes	Unsure but leaning towards yes
#11	34	White British	South West	£35,000–£84,999	1	13.5 mos	Yes definitely	Yes definitely
#12	39	White British	East Midlands	£35,000–£84,999	2	13.5 wks	Unsure but leaning towards yes	Unsure but leaning towards yes
#13	33	Asian or Asian British: - Pakistani	Greater London	£35,000–£84,999	1	6 wks	Yes definitely	Yes definitely
#14	38	White British	South East	Under £34,999	2	5 mos	Unsure but leaning towards no	Unsure but leaning towards no
#15	32	White: - Other white background	East of England	Under £34,999	1	12 mos	Yes definitely	Yes definitely
#16	42	White British	East of England	£35,000–£84,999	1	13.5 mos	Yes definitely	Yes definitely
#17	32	White British	South West	Under £34,999	1	12 mos 3 wks	Unsure but leaning towards no	Unsure but leaning towards yes
#18	34	Chinese	Greater London	£85,000 and over	2	11 wks	Unsure but leaning towards yes	Unsure but leaning towards yes
#19	25	Mixed:- White and Black Caribbean	West Midlands	£35,000–£84,999	2	12 wks	Unsure but leaning towards yes	Unsure but leaning towards yes

### 4.1 Quantitative findings - acceptability of a future COVID-19 vaccination

A high proportion of survey participants reported that they would definitely accept or were unsure but leaning towards accepting a COVID-19 vaccine for themselves (*Yes, definitely* 55.8%, n = 699; *Unsure but leaning towards yes* 34.3%, n = 429) and their child/children (*Yes, definitely* 48.2%, n = 604; *Unsure but leaning towards yes* 40.9%, n = 512) (Table 3). <1 in 10 participants reported that they were unsure but leaning towards rejecting or would definitely reject a COVID-19 vaccine for themselves (*Unsure but leaning towards no 6.2%, n = 78; No, definitely not 3.7%, n = 46*) and their child/children (*Unsure but leaning towards no 7.4%, n = 93; No, definitely not 3.4%, n = 43*).

**Table 3:** Self-reported indication of COVID-19 vaccine acceptance among parents and guardians with a child aged 18 months or under.

**Question**		**Frequency (%)**
If a new coronavirus (COVID-19) vaccine became available would you accept the vaccine for you child/children?		
	Yes, definitely	604 (48.2)
	Unsure but leaning towards yes	512 (40.9)
	Unsure but leaning towards no	93 (7.4)
	No, definitely not	43 (3.4)
	*Total*	*1252 (100)*

If a new coronavirus (COVID-19) vaccine became available would you accept the vaccine for yourself?		
	Yes, definitely	699 (55.8)
	Unsure but leaning towards yes	429 (34.3)
	Unsure but leaning towards no	78 (6.2)
	No, definitely not	46 (3.7)
	*Total*	*1252 (100)*

Participants were more likely to say that they would definitely accept or were leaning towards accepting a COVID-19 vaccine for themselves (mean = 2.42, SD = 0.768) than for their child/children (mean = 2.34, SD = 0.761), *t (1251) = 7.636p < .001*.

### 4.2 Survey open-text and interview findings: Main reasons for accepting the vaccine

Of the survey participants that stated they would definitely accept or were unsure but leaning towards accepting a COVID-19 vaccine for themselves (n = 1128), 897 gave an open-text reason (79.5%). Of the 1116 participants that stated they would definitely accept or were leaning towards accepting a COVID-19 vaccine for their child, 925 gave an open-text reason (82.9%).

Most interviewees said that they would definitely accept or were unsure but leaning towards accepting a COVID-19 vaccine for themselves (n = 18) and their children (n = 16) in their survey responses (see Table 2). Interestingly, one interviewee (#19) that was leaning towards accepting the vaccine at the time of completing the survey discussed that she was leaning towards refusing the vaccination (for herself and her child) at the time of interview.

**Fig. 1:**
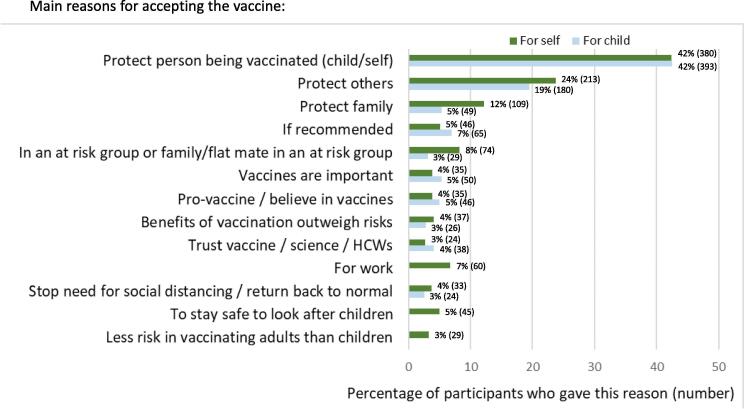
Main reasons for accepting the vaccine.

The following reasons were given for COVID-19 vaccine acceptance for self and for child/children, in order of how often they were mentioned by survey participants and importance to interviewees (see Fig. 1 for the main reasons for accepting the vaccine).

#### 4.2.1 To protect self and others

Of survey participants expressing positive intentions to vaccinate and leaving an open-text response, the most prevalent reason was to provide protection from COVID-19 to the person being vaccinated (*for self*: 42.4%, n = 380; *for child*: 42.5%, n = 393), followed by protecting other people (*for self*: 23.7%, n = 213*; for child*: 19.5%, n = 180), including family members (*for self:* 12.2%, n = 109*; for child*: 5.3%, n = 49). Participants also reported that they would vaccinate to protect someone known to them in a risk group for COVID-19 (*for self*: 8.2%, n = 74; *for child*: 3.1%, n = 29). 5.0% (n = 45) of survey participants specifically mentioned that they wanted to receive the vaccine to stay healthy to look after their child/children. Interviewees also corroborated these findings. They also highlighted that vaccinating would make them feel safer in visiting older family members.

*‘…. for us, I definitely want that. I'd want us protected as a family. You know, my parents, his [partner’s] parents are ageing, so we'd be going to see them, and I don’t want to put them at risk. So, certainly, I think it's really important.’ (Interview #13)*

Several survey participants cited that they would vaccinate as they were key workers (e.g. involved in health and social care, education and childcare, or the food sector) and in frequent contact with other people (6.7%; n = 60). Health and social care workers particularly noted a need to vaccinate to protect themselves and ‘at risk’ patients and clients.

#### 4.2.2 Vaccination as a route to ‘returning to normal life’

For several interviewees, the availability of a vaccine was viewed as the only way of ending social-distancing measures and returning to normal life. Interviewees talked about lockdown as being financially unsustainable, and detrimental to physical and mental wellbeing and children’s educational and social development. Interviewees that were shielding during the pandemic were particularly keen to accept a future vaccine, with one parent who had looked into entering a COVID-19 vaccine trial saying they ‘would be first at the door’.

Survey participants also indicated that they would be vaccinated (3.7%; n = 33) and get their children vaccinated (2.6%; n = 24) as a means of stopping the need for social distancing and returning to normality.

*‘…society can't go back to normal until most of us are immune. A vaccine is the quickest way to achieve that’ (Survey participant #346).*

*‘I believe in the importance of all recommended vaccinations for my child anyway and worry about her [participant’s daughter] contracting COVID-19. I also worry about her [participant’s daughter] losing out on normal experiences such as seeing relatives and friends, going to classes and preschool etc. if we cannot manage the pandemic.’ (Survey participant #857)*

#### 4.2.3 Trust and belief in the importance of vaccination

Survey participants indicated that they would receive the vaccine for themselves (5.1%; n = 46) or their child/children (7.0%; n = 65) if it was recommended by the government or healthcare workers. Survey participants cited their belief in the importance of vaccination as a reason to receive the vaccination themselves (3.9%; n = 35) or to vaccinate their children (5.4%; n = 50).

*‘I trust that if vaccines are approved for general use and recommended by NHS, they are safe.’ (Survey participant #83)*

A proportion of survey participants specifically reported trust in vaccinations, science or healthcare workers as a prompt to vaccinate their child/children (4.1%; n = 38) and themselves (2.7%; n = 24).

#### 4.2.4 Less risk in vaccinating adults

Interviewees and survey participants discussed a greater willingness to ‘risk’ receiving the vaccination for themselves than for their children due to safety concerns. Survey participants also reported the perception that it was less risky to vaccinate adults than children (3.2%; n = 29) as the vaccine trials were conducted in adults.

*‘I feel safer having the vaccine as an adult as trial would be based on adults’ (Survey participant #370)*

### 4.3 Survey open-text and interview findings: main reasons for not accepting the vaccine

Of the participants that stated they would definitely reject or were unsure but leaning towards rejecting a COVID-19 vaccine for themselves (n = 124), 100 left an open-text reason (80.6%). Of the 136 participants that stated they would definitely reject or were leaning towards rejecting a COVID-19 vaccine for their child, 124 (91.2%) gave an open-text response. Three interviewees reported that they were unsure but leaning towards rejecting a COVID-19 vaccine for their child/children. One interviewee (#14) was leaning towards rejecting a COVID-19 vaccine both for themselves and their child (see Table 2).

**Fig. 2:**
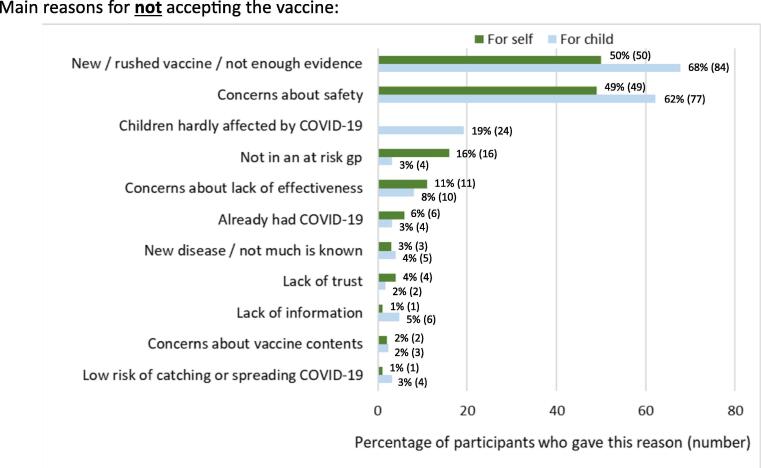
Main reasons for not accepting the vaccine.

The following reasons were given for COVID-19 vaccine rejection by survey participants and interviewees (see Fig. 2 for the main reasons for not accepting the vaccine).

#### 4.3.1 Vaccine safety and effectiveness concerns

The most common concerns expressed by survey participants about a COVID-19 vaccine were around vaccine safety (*for self*: 49.0%, n = 49; *for child*: 62.1%, n = 77) and effectiveness (*for self*: 11.0%, n = 11; *for child*: 8.1%, n = 10), which were largely prompted by the newness and rapid development of the vaccine (*for self*: 50.0%; n = 50; *for child*: 67.7%; n = 84), and the newness of COVID-19 (*for self*: 3.0%, n = 3; *for child*: 4.0%, n = 5). Participants were worried that the development process might be too rapid, not allowing enough time for adequate testing to confirm the vaccine’s short and long-term safety, and also vaccine effectiveness.

These concerns were echoed by interviewees who had reservations around how a vaccine developed in a fraction of the usual timeline for vaccine development could be confirmed as safe.

*‘I'm very pro-vaccines in general, but my main concern was as long as it hasn't been rushed through. As long as we know it' safe and all the proper tests have been done, and, you know, when you sort of heard things in the news like well, it's been 18 years and they haven't developed a SARS vaccines, or whatever it was that was sort of banded around yesterday, it sort of makes you go, why is that? So, I think that if a vaccine was to come out like this one could do in a hurry it need to have associated information to how this has been able to happen so quickly.’ (Interviewee #19)*

Some survey participants and interviewees wanted to ‘wait-and-see’ if the vaccination was safe before being vaccinated themselves, or getting their children vaccinated. One interviewee discussed preferring for her family to remain in lockdown for longer (i.e. living for a longer duration with the restrictions implemented on 23rd March 2020), to allow more time for vaccine development and safety testing. This interviewee acknowledged this situation as not a financially viable option for those unable to work from home.

#### 4.3.2 Children less at risk of COVID-19

Some survey participants expressed a lack of benefit to vaccinating their children, citing that children are hardly affected, are at lower risk of severe COVID-19 infection than adults (19.4%, n = 24), and less likely to catch or transmit COVID-19 (3.2%, n = 4). Participants and interviewees discussed the decision to vaccinate their children in terms of balancing potential advantages and disadvantages. The perception that children are less at risk of COVID-19 was combined with safety concerns as a reason not to vaccinate.

*‘Any vaccine for covid-19 will have been produced in a huge hurry, possibly bypassing some of the normal processes and procedures that ensure safety. As small children have a very low risk of being seriously ill from the virus, I'm not sure I would risk it. I would encourage my mum, who is 70 with underlying health conditions, to take it though as the risk /reward is different.’ (Survey participant #1095)*

Several survey participants indicated that they would be more likely to get their child/children vaccinated if this was beneficial in reducing COVID-19 spread to older people and clinical risk groups.

#### 4.3.3 Low perception of risk

Some survey participants indicated not needing the vaccine as they (16.0%, n = 16) and their child/children (3.2%, n = 4) were ‘fit and healthy’ and not in an at-risk group. 2% of participants (n = 2) indicated that it was better to prioritise ‘at-risk’ groups for vaccination.

#### 4.3.4 Already had COVID-19

A small number of survey participants indicated that they would not accept a COVID-19 vaccine for themselves or their child/children as they (6.0%, n = 6) or their child (3.2%, n = 4) had already had COVID-19.

#### 4.3.5 Need for transparency to make an informed vaccine decision

To be able to make an informed choice about vaccination, parents expressed a need for transparent information on vaccine development, vaccine efficacy and vaccine safety. A lack of vaccine information at present was given as a reason not to accept the vaccine for a minority of survey participants for their children (4.8%, n = 6) and for themselves (1.0%, n = 1). One interviewee also discussed that the information should come from trusted sources such as healthcare professionals, the NHS and Public Health England.

*‘…if the NHS is telling you something you’re more likely to believe it, whereas if it’s a politician, you know, you’ve got much more incentive to think, “Well, are they telling the truth?” (Interviewee #17)*

#### 4.3.6 Lack of trust

A small number of survey participants stated a lack of trust in vaccinations, science or the medical profession as a reason to not accept a vaccine (*for self*: 4.0%, n = 4; *for child*: 1.6%, n = 2).

### 4.4 Quantitative findings – Factors associated with COVID-19 vaccine rejection for self

A forward stepwise logistic regression analysis was performed with a dichotomised version of the self-vaccination against COVID-19 variable as the discrete variable. *Age*, *household income*, *ethnicity*, *location* and *employment* were included as predictive variables. The final model included three predictor variables (*household income*, *employment* and *ethnicity*) and significantly predicted ‘Likely to reject’ (omnibus chi-square = 50.225, df = 7, *p* < .001). *Age* and *location* did not significantly predict ‘Likely to reject’, as such they are excluded from the model. The Hosmer-Lemeshow test demonstrates that the model adequately fits the data chi-square = 4.239, df = 7, *p* = .752. Table 4 gives coefficients and the Wald statistic, odds ratio and associated degrees of freedom for each of the predictor variables.

**Table 4:** Predictors of COVID-19 vaccine acceptance for self.

	**Univariable analysis**			**Multivariable analysis**		
**Variable**	**Sig (*p*)**	**OR**	**95% CI**	**Sig (*p*)**	**OR**	**95% CI**
**Age**	**0.008**	**–**	**–**	**0.559**	**–**	**–**
18–23	0.017	3.575	1.257–10.165			
24–30	0.004	1.860	1.216–2.844			
31–36 †	–	–	–			
37–42	0.710	0.899	0.514–1.574			
43+	0.439	1.634	0.471–5.669			

**Household income**	**<0.001**	**–**	**–**	**<0.001**	**–**	**–**
Low Income <£35,000 (n = 242)	<0.001	2.535	1.677–3.832	0.002	2.087	1.317–3.307
Medium Income £35,000–£84,999 (n = 622)†	–	–	–	–	–	–
High Income >£85,000 (n = 260)	<0.006	0.363	0.177–0.744	0.005	0.352	0.170–0.729

**Employment**	**<0.001**	**–**	**–**	**0.033**	**–**	**–**
Working full-time (n = 628)†	–	–	–	–	–	–
Working part-time (n = 373)	0.802	0.943	0.596–1.491	0.229	0.739	0.452–1.209
Homemaker (n = 103)	<0.001	3.025	1.784–5.130	0.038	1.888	1.035–3.445
Student (n = 10)	0.006	5.673	1.656 –19.436	0.311	2.076	0.506–8.524
Unemployed (n = 10)	0.009	5.042	1.504–16.903	0.224	2.474	0.575–10.651

**Ethnicity**	**0.017**	**–**	**–**	**0.010**	**–**	**–**
White (n = 1070)†	–	–	–	–	–	–
BAME (n = 54)	0.010	2.295	1.216–4.333	0.010	2.733	1.270–5.885

† Reference category.

Participants in the lower household income bracket (<£35,000) were almost twice (OR: 2.08, 95%CI: 1.31–3.3) as likely to reject a COVID-19 vaccine than participants with a medium household income (£35,000-£84,999). Those in the highest income bracket (>£85,000) were almost three times (OR: 0.35, 95%CI: 0.17–0.73) as likely to accept the vaccine as middle-income bracket participants (£35,000-£84,999).

Participants that self-reported as Black, Asian, Chinese, Mixed or Other ethnicity were 2.7 times (95%CI: 1.27–5.87) more likely to reject a COVID-19 vaccine than White British, White Irish and White Other participants. There was also some indication that those that identify their employment as *homemaker* were more likely to reject the vaccination than those in full time employment or on parental leave from full time employment.

### 4.5 Quantitative findings – Factors associated with COVID-19 vaccine rejection for child

A forward stepwise logistic regression analysis was performed with a dichotomised version of the child vaccination against COVID-19 variable as the discrete variable. *Age*, *household income*, *ethnicity*, *location*, *employment* and *number of children* were included as predictive variables. The final model included three predictor variables (*income*, *ethnicity* and *number of children*) and significantly predicted ‘Likely to reject’ (omnibus chi-square = 37.896, df = 6, *p* < .001). *Age*, *location* and *employment* did not significantly predict ‘Likely to reject’, as such they were excluded from the model. The Hosmer-Lemeshow test demonstrates that the model adequately fits the data chi-square = 1.502, df = 6, *p* = .958. Table 5 gives coefficients and the Wald statistic and associated degrees of freedom and probability values for each of the predictor variables.

**Table 5:** Predictors of COVID-19 vaccine acceptance for child

	**Univariable analysis**			**Multivariable analysis**		
**Variable**	**Sig (*p*)**	**OR**	**95% CI**	**Sig (*p*)**	**OR**	**95% CI**
**Employment**	**<0.001**			**0.201**		
Working full-time (n = 628)†	**–**	**–**	**–**			
Working part-time (n = 373)	0.923	1.021	0.666–1.568			
Homemaker (n = 103)	<0.001	2.561	1.511–4.340			
Student (n = 10)	0.074	3.377	0.891–12.805			
Unemployed (n = 10)	<0.001	8.684	2.829–26.660			

**Household income**	**<0.001**			**0.001**	**–**	**–**
Low Income <£35,000 (n = 242)	<0.001	2.291	1.521–3.451	0.007	1.826	1.178–2.830
Medium Income £35,000–£84,999 (n = 622)†	–	–	–	–	–	–
High Income >£85,000 (n = 260)	0.094	0.614	0.347–1.087	0.079	0.596	0.334–1.062

**Number of children**	**<0.001**			**0.004**	**–**	**–**
1 (n = 507)†	–	–	–	–	–	–
2 (n = 447)	0.289	1.249	0.828–1.882	0.409	1.206	0.773–1.880
3 (n = 133)	0.014	1.955	1.146–3.335	0.083	1.677	0.935–3.009
4 or more (n = 37)	<0.001	4.250	2.044–8.836	<0.001	4.054	1.843–8.914

**Ethnicity**	**0.038**			**0.009**	**–**	**–**
White (n = 1,070)†	–	–	–	–	–	–
BAME (n = 54)	0.027	2.046	1.087–3.852	0.009	2.549	1.259–5.159

†Reference category.

Similarly, to accepting a novel COVID-19 vaccination for themselves, participants that self-reported as Black, Asian, Chinese, Mixed or Other ethnicity were 2.74 times (95%CI: 1.35–5.57) more likely to reject a COVID-19 vaccine for their child than White British, White Irish and White Other participants. This finding was also found for income, with participants in the lower household income bracket (<£35,000) being 1.8 times (95%CI: 1.17–2.82) as likely to reject a COVID-19 vaccine for their child than participants with a medium household income (£35,000-£84,999). Participants with more than four children were also found to be around four times (OR 4.13; 95%CI: 1.873–9.104) more likely to reject the vaccination for their children than those with only one child, however, caution should be taken with this finding due to the small sample size.

## 5 Discussion

In this multi-methods study, the majority of parents and guardians reported they would definitely accept or were unsure but leaning towards accepting a COVID-19 vaccine for themselves and their child/children.

Attitudes towards a COVID-19 vaccine appear more positive in our study (in terms of the proportion of participants reporting they would definitely accept or were leaning towards accepting vaccination) than those reported in surveys conducted with adults in France, Germany, Italy, Portugal, the Netherlands and the US [11], [12], [13], and comparable to reports from Denmark and Australia [12], [14]. However, this could be due to differences in the questions asked; highlighting the need for cross-country surveys and consistency in the wording of questions. These surveys indicate that the difference in vaccination acceptance ranges greatly between countries, from around 62% in France to 80% in Denmark and the UK [12], and are reflective of trust in vaccines and health systems more broadly, and in governments. More positive attitudes towards COVID-19 vaccination in our study appear to reflect the high level of parent trust in vaccines in the UK, and trust in the NHS.

The main reasons, or ‘pros’ for vaccinating, were to protect the individual being vaccinated against COVID-19, followed by protecting others from the disease. Uncertainties around vaccine safety, effectiveness, and the benefit of vaccinating children were the most prevalent reasons given for COVID-19 vaccine refusal. Safety and effectiveness concerns were prompted by fears that the accelerated vaccine development process, considered politically motivated, could result in cutting corners. Comparably, previous research also suggests that people consider older vaccines safer than newer ones [15], [16], and highlights the importance of transparency in communicating about the vaccine development process and of vaccination safety testing.

When a COVID-19 vaccine is launched, should any safety signals or safety concerns arise, it will be key for government and public health officials to reassure the public with transparency, action, accountability and timeliness in order to avoid any detrimental impact of the new vaccine on the current national immunisation programme. The dengue vaccine controversy in the Philippines highlights how safety concerns around a newly launched vaccine, Dengvaxia, can derail a successful national immunisation programme leading to drops in childhood vaccine uptake, and even spill over to other health care treatments [17]. A number of safety scares have also derailed vaccine programmes in the past, including safety concerns around the HPV vaccine in Japan which spread globally [18].

Other surveys have identified that views on COVID-19 vaccine acceptance are influenced by vaccine efficacy and perceptions of disease risk. In a cross-sectional survey conducted in Indonesia, Harapan et al [19] found that 93.3% of participants would receive a COVID-19 vaccine that was 95% effective but only 67.0% of participants would accept a 50% effective vaccine. The authors also found that acceptance was higher amongst participants that considered themselves at greater risk of COVID-19 infection.

In our study, lower levels of vaccine acceptance for children were discussed by parents in terms of children being at lower-risk of COVID-19. Evidence suggests that children have lower susceptibility to COVID-19 [20], very few children develop severe COVID-19 (even if they are in a clinical risk group), and their role in the transmission of COVID-19 is unclear [21]. Similarly with influenza vaccination, parents and guardians in our study more often considered the perceived benefits to their child, rather than societal benefits, as a reason to vaccinate their children [22].

### 5.1 Acting now to prevent inequalities in COVID-19 vaccine uptake

We found that participants of Black, Asian, Chinese, Mixed or Other ethnicity were more likely to reject a COVID-19 vaccine for themselves and their child/children compared to White participants (i.e. White British, White Irish or White Other). Comparably, lower levels of COVID-19 vaccine acceptability in Black African and lower income groups have been reported in US based surveys [13]. This is of concern given the evidence that Black, Asian and minority ethnic (BAME) groups and people living in the most deprived areas are at higher risk of acquiring COVID-19 infection and at increased risk of death from COVID-19 [21]. Inequalities in vaccination uptake amongst BAME communities [23], [24], [25], [26] and lower income groups [26], [27] already exist for certain vaccines and a concerted effort must be made to understand factors affecting vaccine acceptance and address potential ethnic inequalities in uptake of a future COVID-19 vaccine. We were unable to explore further reasons for ethnic differences in COVID-19 vaccine acceptance due to insufficient representation of ethnic minority groups in writing open-text responses and agreeing to participate in interviews.

## 6 Strengths and limitations

The quality of the study was enhanced through the use of a multi-methods approach in which interview and open-text responses were used to develop insight into factors underpinning quantitative responses. Our study took place at the height of the COVID-19 pandemic in England and a survey repeated now that we are ‘past the peak’ of COVID-19 cases and deaths and lockdown has been eased may yield different responses. This has already been indicated in the second wave of a large European survey looking at COVID-19 vaccine attitudes [28]. This highlights the need for longitudinal studies to measure the acceptability of a COVID-19 vaccine at different intervals.

Our recruitment strategy achieved a high number of responses (n = 1252). Although largely representative geographically, our sample lacked representability in terms of household income and ethnicity. For example, in relation to ethnicity, according to the 2011 census 85% of the population in England was White. 94% of our survey participants self-reported as White [29]. Our recording of household income is not directly comparable to UK data from the Office for National Statistics (ONS), as we did not specify if the income value provided by survey participants should be pre or post-tax and benefits, or take into account the number of household occupants. We also captured data for income in banded forms, rather than asking for exact amounts. However, our sample appears to have a much higher median household income (£55,000-£64,999) than the median household income for the UK (£29,600) [30]. This meant that although our sample size was large enough to identify ethnicity and income as determinants of vaccine acceptance, it was not possible to explore differences in vaccination views by these variables when looking at open-text responses.

## 7 Conclusion

The success of COVID-19 vaccination programmes will rely heavily on public willingness to accept the vaccine. The main concerns raised by parents in our study were around the safety and effectiveness of a ‘rushed’ and new COVID-19 vaccine. To alleviate these concerns, there needs to be clear communication and transparency as to how COVID-19 vaccines are developed and tested, and safety and efficacy information.

Opening up a conversation with members of the public at an early stage is key to understanding factors that may affect vaccine acceptability, and developing approaches to allay any concerns. This must happen in parallel with efforts to develop a COVID-19 vaccine.

Importantly, efforts must be made to understand and address factors that may affect COVID-19 vaccine uptake in Black, Asian and ethnic minority groups and lower-income households who are disproportionately affected by COVID-19.

## 8 Author’s contributions

SB, RC, SM-J and PP initiated and designed the study. SB led the survey and interview recruitment. SB and PP conducted the interviews. RC led the design of the survey. RC performed the statistical analysis, with the support of JW. PP performed the analysis of open-text survey responses. SB performed the qualitative interview analysis and prepared the first draft of the manuscript. All authors were involved in the interpretation of findings. All authors participated in manuscript preparation and approved the final manuscript.

## Data availability

The full dataset will be made available to bona fide researchers upon request and agreement by the study team. Further information on the data and access conditions can be found through the LSHTM Data Compass at: https://doi.org/10.17037/DATA.00001862.

## Ethical approval

Ethical approval for this study was granted by the London School of Hygiene & Tropical Medicine Observational Research Ethics Committee (Reference: 21879).

## Declaration of Competing Interest

The authors declare that they have no known competing financial interests or personal relationships that could have appeared to influence the work reported in this paper.
